# The use of kiosks to improve triage efficiency in the emergency department

**DOI:** 10.1038/s41746-023-00758-2

**Published:** 2023-02-03

**Authors:** Michael Jose Joseph, Matthew Summerscales, Saieesha Yogesan, Anthony Bell, Michele Genevieve, Yogesan Kanagasingam

**Affiliations:** 1 School of Medicine, University of Notre Dame, Fremantle, Australia; 2 St John of God Midland Public and Private Hospitals, Midland, Australia; 3 School of Medicine, Curtin University, Bentley, Australia

**Keywords:** Health services, Diseases, Diagnostic markers

## Abstract

Triage is a system used to prioritise patients as they enter the emergency department (ED) based on their need for urgent care. In recent decades, EDs have becoming increasingly overcrowded, leading to longer pre-triage waiting times for patients. E-triage interventions like kiosks have been proposed as a solution to overcrowding. We conducted a literature review into the effectiveness of kiosks in improving triage efficiency. After rigorously searching five biomedical databases and screening candidate articles in Endnote, we identified nine papers pertaining to the introduction of kiosks in emergency departments. Six articles had positive findings—with E-triage interventions improving some aspect of the triage process—such as reducing pre-triage times. Conversely, only three articles reported negative findings, such as low uptake. Consequently, EDs should consider introducing kiosks to complement the current nurse-led triage process and thereby promote better patient outcomes.

## Introduction

At an emergency department (ED), a triage system (process by which a clinician assesses a patient’s clinical urgency), is the vital structure by which all incoming emergency patients are prioritised using a standard triage rating scale based on urgency^1^. The purpose of a triage system is to ensure that the level of emergency care provided is corresponding with clinical criteria. ‘Urgency’ is determined according to the patient’s condition on arrival at the EDs. Internationally, five-tier triage scales have been shown to be a valid and reliable method for categorising people who are seeking assessment and treatment in hospital EDs^2^. Two of the most widely adopted scales are the Manchester Triage Score (MTS) and Canadian Triage & Acuity Score (CTAS). These scales are internationally regarded because they have standardised complaint lists.

Pre-triage waiting times for those patients presenting to the ED can be long during peak hours^2^. Patients presenting to the ED are often required to wait until a trained triage nurse is available to initiate the triage process. When multiple people present simultaneously, this sudden increase in presentations can exceed the triage nurse’s ability to manage flow, resulting in long queuing, that can potentially affect timely, quality care. Previous studies have found a link between ED overcrowding and increased patient mortality^3,4^.

Overcrowded hospital ED are becoming an increasingly relevant problem throughout the world due to long pre-triage waiting times^1^. Inadequate staffing and insufficient resources are factors which contribute to longer waiting times for treatment^3^. This has negatively impacted patients’ confidence in the ability of EDs to treat them and their willingness to return in future emergencies, putting them at risk of increased morbidity and mortality^3,4^. The fundamental issue of a triage identification gap means that patients are not being queued effectively, and that some patients are waiting for long periods of time to receive critical care^4^.

Take for example the United States—where the average ED waiting time increased by 25% in the decade leading up to 2009^5^. This may owe to the fact that the number of annual ED visits per 100 persons increased from 35–40 in the 10 years prior to 2006^5^. Concerningly, ED admissions for patients with chronic conditions increased by 30% from 2007 to 2012^5^. These figures highlight how managing ED triage is an increasingly relevant issue which has meaningful impacts on patients’ health outcomes.

In recent years, this issue has been placed under the spotlight, with numerous solutions proposed to shorten ED waiting times and improve patient outcomes. While some solutions focus on improving management systems, multiple proposals incorporate digital health—namely, the use of self-service kiosks, to assist with patient triage in the ED^5–13^.

Kiosks are freestanding devices which resemble ATMs. Patients are prompted to answer algorithm-based questions which allow ED staff to classify their priority level in the queue based on the type and severity of their presenting complaint^5–13^. Most kiosks contain a touch screen interface for ease-of-use^7–10,12^. Some kiosks also have text-to-speech functionality—allowing patients to hear instructions^9^. This data is usually transmitted wirelessly to live feeds monitored by nurses, allowing them to identify patients in need of more immediate care^7^. Transmitted data is securely stored in external locations such as a hospital database or offsite server to protect patients’ confidentiality^7,10^. The goal of these technologies is to support nurse-led manual triaging so that EDs can operate more efficiently^5–13^.

Kiosks attempt to achieve specific outcomes:

i. Have patient specific information entered into the medical information record to decrease unnecessary input by nurses.
ii. Respond to complaint specific questions to help triage nurses prioritise patients in the queue for their formal triage.
iii. Support but not replace the role of the triage nurse.

However, there is little research into whether kiosks have widespread effectiveness. After all, there are many parameters by which we can define improvement—including measures such as waiting times, usability, and pre-triage time.

We sought to generalise these outcomes by conducting a literature review assessing whether there was a negative or positive outcome following the implementation of kiosks in emergency departments. Papers were critically examined via the following four questions:

1. Did these studies examine emergency departments in general or focus on specific presenting conditions like sepsis?
2. What was the proposed solution to improve ED triaging?
3. Did the paper examine ED triaging in ‘normal’ times or in exceptionally challenging periods such as disaster medicine?
4. Were the results of the studies positive or negative?

## Results

**Fig. 1:**
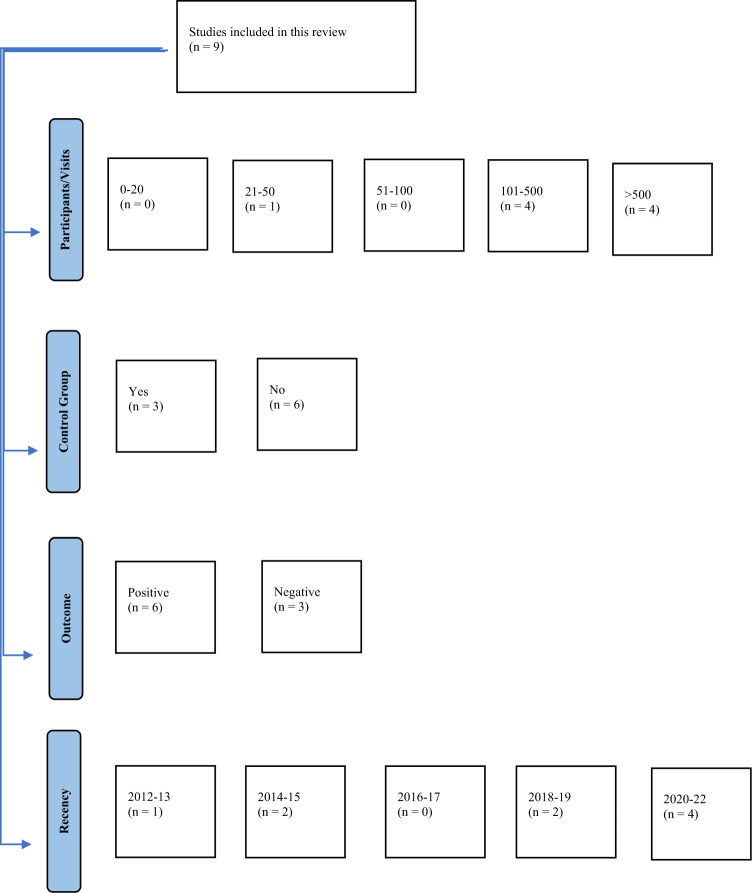
Articles grouped by study characteristics. The number of articles belonging to each category are shown as *n* = . Number of ED visits were considered equivalent to number of participants. Articles deemed to have control groups were those with comparator groups (e.g. nurse-led triage). The positive and negative classification included articles where there was a mix of both outcomes (with the more predominant outcome favoured). Categories for recency of publication were divided into 2-year groups (excluding the last category since the search was completed in early 2022).

Articles were sorted based on four categories—number of participants/ED visits, presence/absence of a control group, outcome, and recency of publication (Fig. 1).

The articles varied in quality, with differences in factors such as the number of participants and recency of publication (Fig. 1).

### Outcomes

A positive outcome was assigned to a paper when the kiosk intervention resulted in an improvement in the triage process (e.g. waiting time, queue length), whereas a negative outcome was assigned when the intervention did not improve the triage process, or even adversely affected it. Some of the studies reported both positive and negative findings. In such instances, the authors assessed the balance of positive to negative findings and assigned the classification to whichever outcome was more prominent.

Six of the selected papers reported positive findings following the implementation of the intervention^5,9–12^, while the remainder reported negative findings (Fig. 1)^7,8,13^.

Mahmood et al. examined how the presence of kiosks affected wait times^5^. Waiting time data was sourced from the National Ambulatory Medical Care Survey (NAMCS) – an annual report which examined ED operations in both not-for-profit and private hospitals across the entire United States (US)^5^. Sampling was probability-based, with data acquired from random visits to random EDs within random hospitals that belonged to random geographic regions of the US^5^. The researchers defined ‘ED wait time’ as the duration in minutes from a patient arriving at the ED to being seen by a health professional (nurse practitioner, physician or physician assistant), and hospital EDs were given a binary classification of ‘yes’ or ‘no’ depending on if they had kiosks^5^. This ‘waiting time’ definition encompasses ED input (time from arrival to placement in a treatment area) and part of throughput (time from end of input to disposition). While there was no control group, direct comparisons of wait times were made between EDs with and without kiosks^5^. Consequently, wait times were determined for an extensive number of hospitals from a wide range of geographical regions servicing a vast array of patient demographics. This study had positive outcomes because EDs which used kiosks were associated with an almost 57% shorter waiting time compared to those without^5^.

Coyle et al. examined the effect of kiosks on patient identification and queuing in a Toronto ED by analysing four outcomes^6^. These were: (1) ‘time-to-first-identification’—the duration in minutes between patient arrival at the ED and identification in the hospital’s Emergency Database Information System (EDIS), (2) ‘usability rate’ - the percentage of ambulatory patients presenting to ED who used a kiosk during intervention weeks, (3) ‘time-to-triage’: the duration between arriving at the ED to completing triage and (4) ‘time-to-MD’: the duration between arriving at the ED to being seen by a physician^6^. Researchers assigned specific intervention weeks where kiosks were present in the ED during the peak hours (10:30–18:30) of weekdays, and control weeks when kiosks were absent^6^. During intervention weeks, the use of kiosks by patients electronically alerted nurses to their arrival and primary complaint, whereas during control weeks patients were identified via the standard nurse-based triage^6^. Across the 10-week study period, data relating to the above four outcomes was gathered from the EDIS^6^. The intervention and control groups had a good mix of males and females, various age groups and a range CTAS scores (patients with CTAS 1 scores were excluded due to their urgency)^6^. The study had positive outcomes because the kiosks significantly reduced time-to-first-identification (intervention was 13.6 min faster; 95% confidence interval (CI) = 12.8–14.5) and had a 97% usability rate across the intervention group^6^. However, similar improvements were not found for time-to-MD and time-to-triage^6^.

Trivedi et al. examined the accuracy of kiosks in assessing patient acuity and predicting hospitalisation^7^. Participants in this study were English speakers, above the age of 16 who entered the ED without the assistance of an ambulance^7^. Patients answered a short algorithm-based questionnaire which generated an algorithm-generated self-triage score (AGST) and were also asked to assess whether they required hospitalisation^7^. Following this, they underwent formal assessment by a triage nurse and were assigned a CTAS score, with need for hospitalisation determined by medical records^7^. Comparison of the self-assisted kiosk scores and nurse-led standard was used to generate the results. The study had primarily negative outcomes because only 17% of patients who predicted hospitalisation were hospitalised and because 73% of patients under- or over-estimated acuity when answering a kiosk questionnaire^7^. However, the questionnaire demonstrated high sensitivity (percentage of true positives) for some serious conditions like cardiorespiratory issues (50%)^7^.

Ackerman et al. examined the effectiveness of introducing a UTI kiosk into four Californian EDs after its successful implementation in an urgent care centre (UCC)^8^. Introduction of the kiosks into the EDs was largely unsuccessful when trialled in adult women (18-64) presenting with UTI symptoms who did not have recurring complaints, such that the devices were removed in three of the four hospitals^8^. Researchers used a sociotechnical analysis based on actor-network theory to explore the reasons why the ED kiosk implementation was largely unsuccessful^8^. Semi-structured interviews of ED staff were conducted to gather their thoughts on these devices^8^. Staff attributed unsuccessful kiosk implementation to factors such as the positioning of the kiosks in areas of the ED which limited the likelihood of patient use, nurse reluctance to refer patients to the kiosks, a low rate of uptake for the UTI kiosks because of highly specific eligibility criteria, and reluctance of clinicians to accept kiosks because of the device’s perceived inability to detect more severe complications^8^. Thus, this study had negative outcomes because it was found that the UTI kiosk did not work well in an ED environment due to range of factors independent of its technological functionality.

Sinha et al. examined the use of kiosks in streamlining the data entry process in EDs^9^. In this study, patients from a paediatric hospital in a low socioeconomic, inner-city, predominately Latino area who arrived at the ED during off-peak hours (10:00–15:00) were assessed by triage nurses^9^. Those determined to be non-urgent (Emergency Severity Index score of 4 or 5) were eligible for participation^9^. Participants were then randomly assigned to kiosk triage (via a bilingual (English and Spanish) audio-assisted device) or nurse-led triage^9^. For most patients, data entry was completed by parents/guardians, but in some cases the child themselves or a sibling/friend completed the questionnaire^9^. Data on the entry times were collected over an 11-week period. This study had primarily positive outcomes because kiosks users were able to enter medical history data significantly faster than nurses could acquire it using standard methods (mean time was 96 s and 127 s respectively)^9^. However, time-to-enter data was inversely related to education level in the kiosk group (*r* = −0.264), but not in the control (*r* = 0.05)^9^.

Boltin et al. simulated an influx of patients to an ED following a historical chemical mass casualty incident (MCI) in which a train derailment led to a large group of people being exposed to chlorine gas^10^. They examined the usefulness of an AI-based kiosk system in accurately assessing MCI patients’ exposure and suggesting appropriate actions^10^. Two groups of participants were recruited from the University of Southern California (USC) nursing programme (predominately young adult females)^10^. The first group was provided with cards which prompted them to enter specific details (sourced from historical records of the incident) into the kiosks—including their symptoms and location they fell ill due to chlorine exposure^10^. The other group acted as a control by presenting with issues (as detailed on the card) unrelated to the MCI^10^. All patients entered the ED with a unique identifying barcode wristband and their detail card. If the primary triage nurse classified them as ‘immediate’, they were taken away for care, but were otherwise sent to a kiosk^10^. The device used an Irritant Gas Syndrome Agent (IGSA) algorithm as part of the Emergency Department Informatics Computational Tool (EDICT) to retrieve information from the patients like symptoms and spatial proximity to the exposure source^10^. From this, the AI made a judgement on the patient’s exposure level (exposed, potentially exposed, or unexposed) and subsequent recommended action (exit—send for retriaging, monitor—for symptom progression, or urgent—seek immediate care)^10^. Nurses then conducted their own independent assessment of the patient and determined exposure level and action. Comparison between the nurse and AI judgements formed the basis of the results^10^. The study had positive outcomes because almost all the participants were able to complete the kiosk query in under 3.5 min and nurses agreed with the systems choice of exposure level around 92% of the time, and suggested action over 84% of the time^10^. However, these results may not be applicable to the untrained public because the participants were health professionals.

Alishahi Tabriz et al. examined the effect of 20 interventions—including kiosks, to reduce overcrowding in United States EDs^11^. As with Mahmood et al., data was acquired from NHAMC survey results between 2007-2015^11^. Information from over 250000 ED visits was collated, with patients representing a thorough mix of age, sex, ethnicity, income status and acuity level^11^. The researchers had permission to access restricted sections of the survey, which contained questions on ED operations that were used to create the study’s dependent variables. Among those considered, boarding time and waiting time were the two major performance measures relevant to kiosks^11^. The former was defined as the time from being admitted to departure from the ED, while the latter was defined as the time between entering the ED and being evaluated by a health professional^11^. Binary outcomes were considered, with prolonged boarding time being classified as anything exceeding 2 h (otherwise not prolonged) and prolonged waiting time being classified as anything exceeding 15 min (otherwise not prolonged)^11^. The study had positive kiosks outcomes because the patients visiting EDs with self-check-in devices had decreased odds of experiencing prolonged waiting times (odds ratio (OR) = 0.56, 95% CI = 0.41–0.83) and boarding times (OR = 0.55, 95% CI = 0.35–0.85) than patients visiting EDs without kiosks^11^.

Eijk et al. examined the effectiveness of a computer assisted patient-self-triage device (ca-ISET) against a trained triage assistant standard (adaptation of MTS for eye conditions) at the ophthalmological ED of Rotterdam Eye Hospital (REH)^12^. ca-ISET was developed from a pre-triage pen and paper tool^12^. The app had 11 iterations in which progressive changes were made to order of response, questions asked, and specific app modules to improve its effectiveness^12^. The algorithm focused on the cause of the complaint (e.g. chemical injury or foreign bodies), and symptoms (e.g. pain or vision loss)^12^. Three versions (1.4,1.6 and 1.11) were tested in this study on adult patients who spoke Dutch and did not have recurring eye complaints. Patients were recruited only when the researcher was present in the ED, and after having been seen and colour-coded (assigned a triage level) by the triage assistant (study had no control)^12^. Triage colour levels were based on maximum allowed waiting time—red (0 min), orange (10 min), yellow (30 min) and green (120 min)^12^. Participants represented a good mix of male and females and a broad age range (27-82)^12^. The study used a binary classification system of high-urgency patients (maximum allowed waiting time of 30 min) and low-urgency patients (30–120 min waiting time)^12^. Comparisons between the triage assistant’s urgency-grading (converted from colour-coding) and the ca-ISET urgency-grading were used to generate the results^12^. This study had positive results because the sensitivity and specificity increased between version 1.4 (sensitivity = 0.67; 95%CI: 0.38–0.95; specificity = 0.69;95CI = 0.42–0.96) and 1.11 (sensitivity = 0.80; 95%CI: 0.68–0.92; specificity = 0.78;95CI = 0.62–0.95)^12^. Additionally, the accuracy increased from 0.69 in version 1.4 to a high level of 0.80 in version 1.11^12^.

Dickson et al. examined the agreement of patient e-triage scoring with gold standard nurse-led MTS scoring^13^. This retrospective study focused on 2 UK emergency departments and involved over 25000 adult ambulatory patients with a diverse mix of sex, age and presenting complaints^13^. There was no control group—rather, each participant conducted their own e-triage assessment and were then subsequently assessed by a nurse-led MTS triage (paired triage)^13^. The kiosk algorithm assessed the patients responses and assigned a priority level that was based on a five-tier urgency scale compatible with the MTS—with Priority 1 patients requiring immediate physician care and Priority 5 patients being nonurgent^13^. Low acuity/urgent and high acuity/non-urgent were respectively defined as MTS red or orange/priority 1 or 2 (e-triage) and MTS green/blue or priority 4 or 5 (e-triage)^13^. Direct comparison of the two triaging processes was used to generate the results^13^. This study had primarily negative outcomes because agreement between e-triaging and nurses was low (weighted kappa = 0.14; 95%CI = 0.14–0.15 and *r* = 0.321)^13^. Additionally, kiosks users were found to under-triage by around 10% and over-triage by almost 60% when compared to the nurse standard^13^. Despite this, there were minor positive findings that the kiosk e-triaging had slightly more specificity for low acuity patients (88.5% vs 80.6%) and substantially more sensitivity from high acuity patients (88.5% vs 53.8%) in comparison to the nurse standard^13^.

The following table provides information on the nature of the software, functionality, and data collection of the kiosks (Table 1). Three articles are absent because they did not provide any details on the kiosk specifications^5,6,11^.

**Table 1:** Comparison of kiosks used in the literature.

Paper	Software	Data Transfer	Functionality/Hardware	Data storage
Trivedi et al.	FluidSurvey software was used to design the device’s questionnaire algorithm (SurveyMonkey Canada Inc., Ottawa, Canada).	Wireless Transmission.	Touchscreen device.	Electronic databases linked with patient identifiers.
Ackerman et al. (via Stein, 2011).	UTI kiosk management module developed using Macromedia design software was modelled on telephone-based UTI treatment programmes.	Not specified.	Bilingual touchscreen interface (English and Spanish) with an audio handset and printer (Kiosk Information Systems [KIS], Inc., Louisville, CO).	Printouts of UTI module responses and clinician recommendations were provided to participants.
Sinha et al.	Microsoft Visual C# (Microsoft®, Redmond, VA) and Python programming languages (Python Software Foundation, Beaverton, Oregon) were used to develop the kiosk software.	Not specified.	Touch screen interface and bilingual (English and Spanish) text-to-speech systems were developed using Windows Forms Designer, Microsoft Speech Application Programming Interface and Microsoft Touch Application Programming Interface.	Time-stamped printouts of participants responses were retained by researchers.
Boltin et al.	The IGSA algorithm was developed using EDICT software.	Wireless bidirectional transfer of information occurred between mobile devices and storage servers (client-server model).	Touch screen interface.	Data was stored externally on a data-storage sever and locally on each client device.
Eijk et al.	Software was developed by Delft Dimensions from an ISET pen-and- paper tool.	Not specified.	21-inch Windows touch screen device affixed to a wheeled trolley, with an XML configuration file used to generate the screen display.	Questionnaire responses were electronically logged and converted to an urgency level.
Dickson et al.	Software consisted of a consultation platform which assessed answers to clinical questions template to group patients into one of five triage levels.	Data was transmitted from kiosks to the Hospital Clinical Symptoms Network (HCSN) via a secure messaging system.	Encased 12.9-inch iPad Pro touchscreen attached to desk/wall/bench with socket guards.	Urgent care metadata was feed into the kiosk system from a separate database and completing kiosk information was stored in the Clinical Record System.

## Discussion

The purpose of this review was to locate all English, peer-reviewed research articles from the past 10 years relating to the use of kiosks in emergency department triage. Articles were sourced from five major biomedical databases (Table 3).

These kiosk technologies are part of ‘E-triage’ – which involves the use of computerised systems to prioritise patients as they arrive in the ED. E-triage has been proposed as an intervention to reduce the time taken to complete triage—which is currently nurse-led.

For example, patients who arrive at the emergency department at St John of God (SJOG) Midland Hospital take around 9–10 min to triage—which is much higher than the Australian national standard of 5 min. About 40% of that time is attributed to nurses manually entering patient data into the hospital system. Evidently, E-triage measures which streamline the data entry process could shorten triage time in hospitals like SJOG Midland.

When considering implementing kiosks, hospitals are interested in clarifying various queries about their use. These include:

1. Do the kiosks save ED staff time and confirm demographic information?
2. Do they allow the patients to reliably enter data that would otherwise be filled by a nurse for the purposes of prioritising assessment?
3. Do they provide a questionnaire which enables bedside ED staff to identify the issue(s) of concern more quickly?
4. Is there patient and staff satisfaction with the kiosk implementation?

The articles discussed all addressed some combination of the above four questions.

In general, patients were eligible to use kiosks in the studies if they were:

- Ambulatory
- Cognisant and/or educated enough to accurately enter information
  And ineligible if they were:
- Significantly distressed and requiring immediate care

E-triage efficiency: There are numerous measures which relate to E-triage efficiency. Four major examples from the kiosk articles include^5–13^:

1. Pre-triage time: How long a patient must wait to be triaged followed arrival at the ED.
2. Time- to-enter data: How long it takes for patients to enter the necessary information into a kiosk.
3. Usability: The percentage of eligible patients visiting the ED who opt to use the kiosk.
4. Agreement with nurse-led triage: The percentage of cases in which the kiosk system patient assessment (e.g. triage priority level and recommended action), matched those of the triage nurse.

Table 2 details the measures which each article assessed.

**Table 2:** The types of triage efficiency measure each article assessed.

Article	Measures
Mahmood et al.	None assessed.
Coyle et al.	1,3
Trivedi et al.	4
Ackerman et al.	None assessed.
Sinha et al.	2
Boltin et al.	2,4
Alishahi Tabriz et al.	None assessed.
Eijk et al.	4
Dickson et al.	4

Mahmood et al. found that United States EDs which had implemented kiosks had appreciably shorter waiting times then those without^5^. Reducing waiting times by almost half may be essential in preventing ED overcrowding and thereby reducing odds of patient mortality^3,4^. A large, diverse study like this lends credibility to the idea that kiosk interventions can help reduce triage times in emergency departments. However, the article does point out that factors such as arrival day/time, hospital location, and hospital capacity protocols can also heavily influence waiting times and thus require consideration by hospitals planning to implement kiosks^5^.

There was high usability and drastic improvements in time-to-first-identification following implementation of the Coyle et al. kiosks^6^. Considering that EDs like that at SJOG Midland spend a large percentage of triage time with manual data entry by nurses, the ability of self-service kiosks to streamline the process by several minutes represents a major improvement in triage efficiency. This study highlighted how effective kiosks can be in saving time during triage identification and reinforced how kiosk uptake can be high in EDs. This could be especially important since most EDs do not currently use powerful emergency medical record systems (EMRs) likes Cerner or EPIC, meaning that most of the data collection and recording will be done by triage nurses, in addition to collecting and evaluating waiting risk. Thus, kiosks could capture some of this data and reduce the manual entry time for triage nurses. Other time-saving alternatives such as scannable patient healthcare identity cards and limited mandatory items could also be implemented.

The kiosks assessed in Trivedi et al. were associated with a high degree of misestimation for acuity level and hospitalisation compared to nurse standards^7^. These results reinforce how questionnaire algorithms which are poorly designed can lead to inaccurate patient assessments by the kiosk system. Simple ‘yes-no’ logic may not be sufficient to assign patients to an appropriate triage category^7^. Additionally, the enhanced predictive sensitivity of kiosks for cardiorespiratory conditions is not necessarily useful because, in a clinical context, patients presenting with serious issues should not be directed to the devices and instead receive immediate care.

The sociotechnical study by Ackerman et al. highlighted the non-technological reasons that UTI diagnosis kiosks were removed from a group of EDs^8^. The failure of the kiosks due to factors like lack of nurse motivation and highly specific eligibility criteria demonstrate how technologies which are conceptually sound do not necessarily work in real-world contexts where complex social factors are present. This study was unique among those included in this review because it was the only one which examined how health practitioner satisfaction can affect the implementation of kiosks. Lack of motivation meant ED staff were not enthusiastic about directing patients towards the devices. Furthermore, the specificity of the kiosks for only UTI presentation contributed to their low uptake. This second result suggest that kiosks should be able to assess a wide range of presenting complaints so that they can have broader applicability in EDs.

The bilingual kiosks studied by Sinha et al. were associated with much faster data entry than the nurse-led standard and there was an inverse relationship between education level and entry times^9^. The first result demonstrate how useful kiosk self-triaging can be in enhancing the speed of ED operations—with entry times being reduced by almost 25%. Given that EDs like that SJOG Midland spend almost half of triage time on manual data entry, saving those few minutes via self-check-in kiosks could have significant impacts on triage efficiency. However, the second result alludes to a potential limitation of kiosks—that their efficient use often requires a level of patient education which is not needed in nurse-led triage.

The Boltin et al. study showed promising results for kiosk implementation—with most patients completing the questionnaire in a few minutes and there being a high degree of agreement between the AI system and nurses^10^. Results like this show how kiosks are especially useful in gathering information quickly from a large volume of patients during MCIs (disaster medicine) or other busy periods, when timely pre-triaging is essential to reduce mortality and morbidity. However, this algorithm was specifically designed for a large-scale chlorine exposure event, meaning its applicability to other MCIs is questionable.

The study of crowding interventions by Alishahi Tabriz et al. found that kiosk check-in measures decreased the likelihood of prolonged waiting times and boarding times^11^. The large, diverse data set from which these results were compiled provides strong evidence that kiosks can reduce waiting times. However, caution must be used in interpreting the boarding time results. Although the study presents statistically significant evidence that boarding times are reduced in EDs with kiosks, the size of this effect is questionable since protracted boarding times are thought to be primarily due to access block (waiting >8 h for an inpatient bed to become available)^3^.

They were substantial improvements in accuracy, sensitivity, and specificity (compared to nurse-standard) in updated versions of the ca-ISET devices featured in Eijk et al.^12^. These results emphasise how changes to kiosk algorithm questionnaires can create meaningful improvements in their ability to correctly classify patients triage levels. However, as with Trivedi et al., the applicability of this devices to high urgency patients is questionable since they require more immediate care. Additionally, this study focuses specifically on an ophthalmological algorithm and is therefore not applicable to most presenting conditions. Trials of such computer-assisted tools would need to be conducted in non-specialised EDs to test its usefulness in managing a range of presenting conditions.

The kiosks studied by Dickson et al. showed low agreement with the nurse comparator, but higher specificity for low urgency patients and higher sensitivity for high acuity patients^13^. This first result highlights how kiosks algorithms can fail to accurately assess patients’ priority levels. Rigorous algorithm design is required so that kiosk applications can repeatedly make triage decisions which corroborate nurse assessments. Regarding the low urgency results, the fact that e-triage demonstrated higher specificity than the nurse-standard is promising. However, as with Trivedi et al, and Eijk et al., the usefulness of e-triage’s additional sensitivity for high acuity presentations is questionable because patients like this require urgent care and should not be sent to kiosks. Overall, high levels of disagreements between algorithms and nurses may lead to antagonism from ED staff who feel as though kiosks are determining patients’ presentations to be more urgent than they are, potentially wasting hospital resources.

The fundamental barriers to kiosk implementation pertain to the number of ED visitors who are eligible to use them, social and logistical factors which impede their adoption in EDs, and their ability to accurately assess patient triage levels.

Many patients fail to pass the eligibility/ineligibility criteria for kiosk use because they enter the ED in an immobilised or unconscious state—such as those brought in by ambulance (BIBA), are not conversant in the kiosk language, or do not have the medical literacy required to complete the questionnaire. Owing to the first point, many studies restricted kiosk access to ambulatory patients. However, low eligibility was also due to kiosks only being used for specific conditions like UTIs or eye issues^8,12^. If hospitals are to justify the costs of adding kiosks to their EDs, then perhaps kiosks need to be useful for a wide scope of presentations. The second point about language barriers is a more minor concern and can be overcome with multilingual kiosks, as was done in multiple studies^6,8^. Regarding the third point, Sinha et al.’s finding that parents who were less educated took longer to complete the questionnaire highlights how a certain level of medical literacy is required to efficiently self-triage using kiosks^9^. In cases where a person cannot enter kiosk data themselves, it may fall on nurses or the patient’s family to enter the information on their behalf through the kiosk or from a separate device (e.g. tablet) which is connected to the triage network.

Non-technological factors such as nurse unwillingness to refer patients to kiosks and physicians’ mistrust in kiosk assessments can represent barriers to their successful implementation in EDs^8^. Given the high stakes natures of EDs, it is understandable that health professionals may be reluctant to rely on algorithms for triage classification. Hospital management should therefore emphasise the benefits of kiosks to ED staff and take steps to ensure kiosks are well-designed so that practitioners’ concerns are addressed.

Some studies found discrepancies between nurse and algorithm-base assessment of ED patients^7,13^. Eijk. et al.’s finding that algorithm design can have significant effects on sensitivity and specificity underlines the importance of questionnaire design in accurate patient assessment^12^. Algorithms like those of Trivedi et al. which rely on basic yes-no logic may have trouble detecting more insidious conditions that could otherwise be flagged by triage nurses^7^. This may lead to unnecessary work for ED staff who send home patients that the algorithm flagged as requiring admission. Additionally, as shown in Eijk et al., progressive iterations of algorithms may be needed to enhance the quality of the patient assessment—which is something most articles did not consider. Kiosk designers should seek to base algorithms off internationally accepted triage scales such as MTS and CTAS which have standardised complaint lists, so that the devices are better able to classify patients’ acuity for a range of presentations. Furthermore, kiosks should have minimal technical jargon so that people of all education levels are able to clearly follow the on-screen directions.

The kiosk articles themselves had limitations because many did not explore all the useful questions that hospitals would like answered prior to implementing kiosks into their emergency department. For example—they may have found association with shorter waiting times but failed to examine staff and patient satisfaction with the devices.

In summary, this paper presented some promising evidence that kiosks may be useful in improving ED triage efficiency. However, there are currently too few studies which have examined the effectiveness of this intervention. Further studies with large, diverse participant groups should be conducted across different jurisdictions to strengthen the evidence base and to assess the generalisability of this approach in a global context. Ultimately, kiosks may present an exciting opportunity to decrease the logistical issues of ED triaging.

## Methods

A broad narrative literature review was conducted.

### Databases

Articles were sourced from five databases: Medline (PubMed), EMBASE (Ebsco), CINAHL (Ovid), Scopus (Elsevier) and Web Of Science (Clarivate). Medline was searched via PubMed, while the other databases were accessed through the University of Western Australia’s OneSearch library. Article sorting occurred via the 2020 Prisma guidelines for reviews.

### Dates

Published papers from the last 10 years (2012-2022).

### Search strategy

The researchers used Boolean operators of ‘AND’ and ‘OR’ to capture articles which contained the relevant keywords. Search terms of ‘emergency department’, ‘emergency ward’, ‘emergency room’, ‘emergency room overcrowding’, ‘ED’, ‘ER’, or ‘accident and emergency’ had to occur in conjunction with ‘triage’, ‘triage in the emergency department’ or ‘triage system’ and ‘kiosks’, ‘self-service’, ‘computer interface’ or ‘self-triage’. Each database was searched with slightly different combinations of these keywords according to search suggestions and predefined categories. The extensive number of search terms meant that all relevant papers could be identified. This search strategy returned hundreds of papers, which were then imported into EndNote^TM^ X8 (Clarivate: Philadelphia USA, London United Kingdom) referencing software and sequentially filtered based on relevance. After all the abstracts were read, papers with high relevancy were examined in-depth.

### Inclusion criteria

All articles published in English within the past 10 years which are peer-reviewed and contain an abstract.

### Exclusion criteria

i. Takes the form of an editorial, conference paper or case report.
ii. Does not take place in, or simulate, an ED.
iii. Does not involve the ED triage process.
iv. Does not discuss kiosks as solutions to ED overcrowding/waiting times.

### Shortlisting

**Fig. 2:**
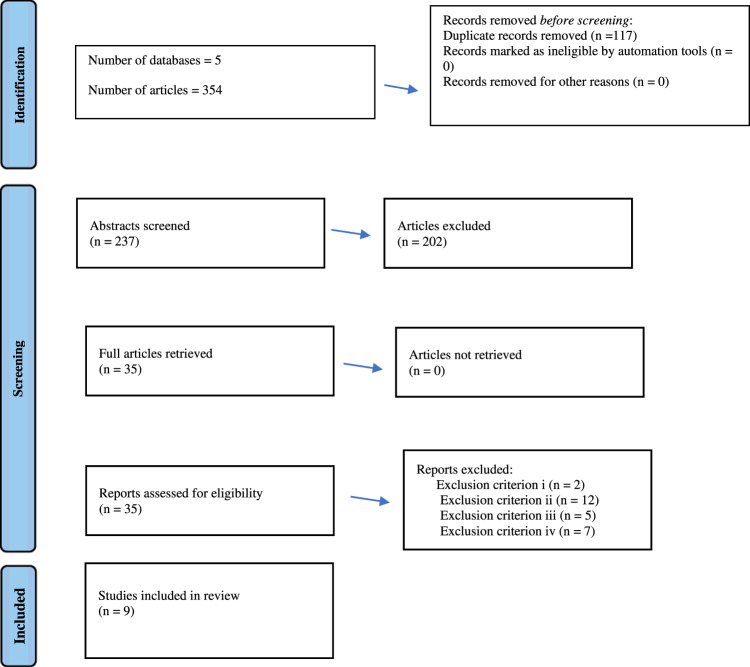
Schematic representation of the shortlisting process (adaptation of the 2020 PRISMA guidelines for reviews). The sequential reduction in the number of articles is shown in descending order. Since there are overlapping search fields in the different databases, a large proportion of the total number of papers were redundant, leading to the removal of duplicates. The authors read the article titles to determine which abstracts were appropriate for reading. The relevancy of the abstracts was used to locate candidate articles for full reading, with the exclusion criteria being applied to these candidates to arrive at a final selection of papers.

Papers which met the inclusion criteria were imported into Endnote^TM^ X8. The imported studies were filtered using an adapted version of 2020 PRISMA guidelines for reviews (Fig. 2, Table 3). Shortlisted papers were assessed via the exclusion criteria until a final selection was made.

**Table 3:** Number of articles returned by database and subsequent shortlisting.

CINAHL PLUS	126
Medline	48
EMBASE	31
Scopus	110
Web Of Science	39
Total number of papers (accounting for duplicates)	237
Number of abstracts selected for in-depth reading	35
Number of papers excluded due to criterion i	2
Number of papers excluded due to criterion ii	12
Number of papers excluded due to criterion iii	5
Number of papers excluded due to criterion iv	7
Final number of papers	9
